# Non-Motor and Motor Features in LRRK2 Transgenic Mice

**DOI:** 10.1371/journal.pone.0070249

**Published:** 2013-07-30

**Authors:** Zoë Bichler, Han Chi Lim, Li Zeng, Eng King Tan

**Affiliations:** 1 Behavioral Neuroscience Laboratory, National Neuroscience Institute, Singapore, Singapore; 2 Duke-NUS Graduate Medical School, Program in Neuroscience and Behavioral Disorders, Singapore, Singapore; 3 Neural Stem Cell Laboratory, National Neuroscience Institute, Singapore, Singapore; 4 Department of Neurology, National Neuroscience Institute, Singapore General Hospital, Singapore, Singapore; National Institute of Health, United States of America

## Abstract

**Background:**

Non-motor symptoms are increasingly recognized as important features of Parkinson’s disease (PD). LRRK2 mutations are common causes of familial and sporadic PD. Non-motor features have not been yet comprehensively evaluated in *LRRK2* transgenic mouse models.

**Objective:**

Using a transgenic mouse model overexpressing the R1441G mutation of the human *LRRK2* gene, we have investigated the longitudinal correlation between motor and non-motor symptoms and determined if specific non-motor phenotypes precede motor symptoms.

**Methodology:**

We investigated the onset of motor and non-motor phenotypes on the *LRRK2^R1441G^* BAC transgenic mice and their littermate controls from 4 to 21 month-old using a battery of behavioral tests. The transgenic mutant mice displayed mild hypokinesia in the open field from 16 months old, with gastrointestinal dysfunctions beginning at 6 months old. Non-motor features such as depression and anxiety-like behaviors, sensorial functions (pain sensitivity and olfaction), and learning and memory abilities in the passive avoidance test were similar in the transgenic animals compared to littermate controls.

**Conclusions:**

*LRRK2^R1441G^* BAC transgenic mice displayed gastrointestinal dysfunction at an early stage but did not have abnormalities in fine behaviors, olfaction, pain sensitivity, mood disorders and learning and memory compared to non-transgenic littermate controls. The observations on olfaction and gastrointestinal dysfunction in this model validate findings in human carriers. These mice did recapitulate mild Parkinsonian motor features at late stages but compensatory mechanisms modulating the progression of PD in these models should be further evaluated.

## Introduction

Parkinson’s Disease (PD), a common neurodegenerative disease, has a complex etiology where both genetic and environmental factors play a role. The diagnosis of PD is essentially based on the motor features that appear relatively late in the time course of the disease, such as resting tremor, rigidity and bradykinesia, by which time more than 70% of the dopaminergic neurons have degenerated [1]. Numerous non-motor symptoms contribute to significant disability in PD [2], [3], [4]. Olfactory loss, rapid eye movements sleep behavior disorder, mood disorders and constipation are commonly described in patients [4], [5]. Increased co-morbidity of non-motor symptoms was associated with greater PD severity and some have even been suggested to be a risk factor for PD as they might precede for years the clinical diagnosis [6], [7], [8]. Understanding the etiopathology of the disease may thus help unraveling specific early biomarkers and would certainly improve the design of future therapies [9], [10], [11].

Mutations of the Leucine-Rich Repeat Kinase 2 (*LRRK2*) gene are the most common causes of sporadic and autosomal dominant PD [12], [13], [14], [15], [16]. LRRK2 is a large protein of 2527 amino acids and multiple protein domains, with pathogenic mutations distributed throughout its length: G2019S is the most common mutation identified, with a prevalence of 13 to 40% depending on the ethnic races. R1441G is the second most common site of pathogenic LRRK2 substitutions. We have previously identified other common genetic variants such as G2385R that are unique to Chinese [17].

Most clinical studies on non-motor symptoms are cross sectional in design, as it is difficult to conduct short-term longitudinal studies on the progression of such phenotypes in humans. Several animal models for PD have been developed, but most of them focus on motor and neuropathological features and they do not recapitulate entirely clinical PD [18], [19], [20], [21], [22], [23], [24], [25], [26], [27], [28], [29], [30], [31], [32]. Recently, Li and collaborators have created a mouse line that sums up the main features of human PD [22]. These mice overexpress the R1441G mutation of the human *LRRK2* gene by means of a Bacterial Artificial Chromosome (BAC). The *LRRK2^R1441G^* BAC transgenic (Tg) mice displayed an age-dependent slowness of movements that begun at 6 months old, and, at 10–12 months of age, the motor deficit was associated with diminished dopamine release and axonal pathology of nigrostriatal dopaminergic projections. These mice had also an increased level of neuroinflammation inducing neurotoxicity [33]. However, the authors did not conduct a thorough evaluation of non-motor features. In addition, motor features were not reported on mice above the age of 12 months. Unpublished data from the Jackson’s laboratory (http://jaxmice.jax.org/strain/009604.html) suggest that the motor features could not be replicated.

To address limitations of current literature, we conducted a detailed longitudinal study of both motor and non-motor features in the *LRRK2^R1441G^* mutant mouse line. Our main hypothesis was that motor symptoms correlate with non-motor symptoms over the course of the disease.

## Results

### Decreased Locomotor Activity in *LRRK2^R1441G^* Tg Aged Mice

**Figure 1:**
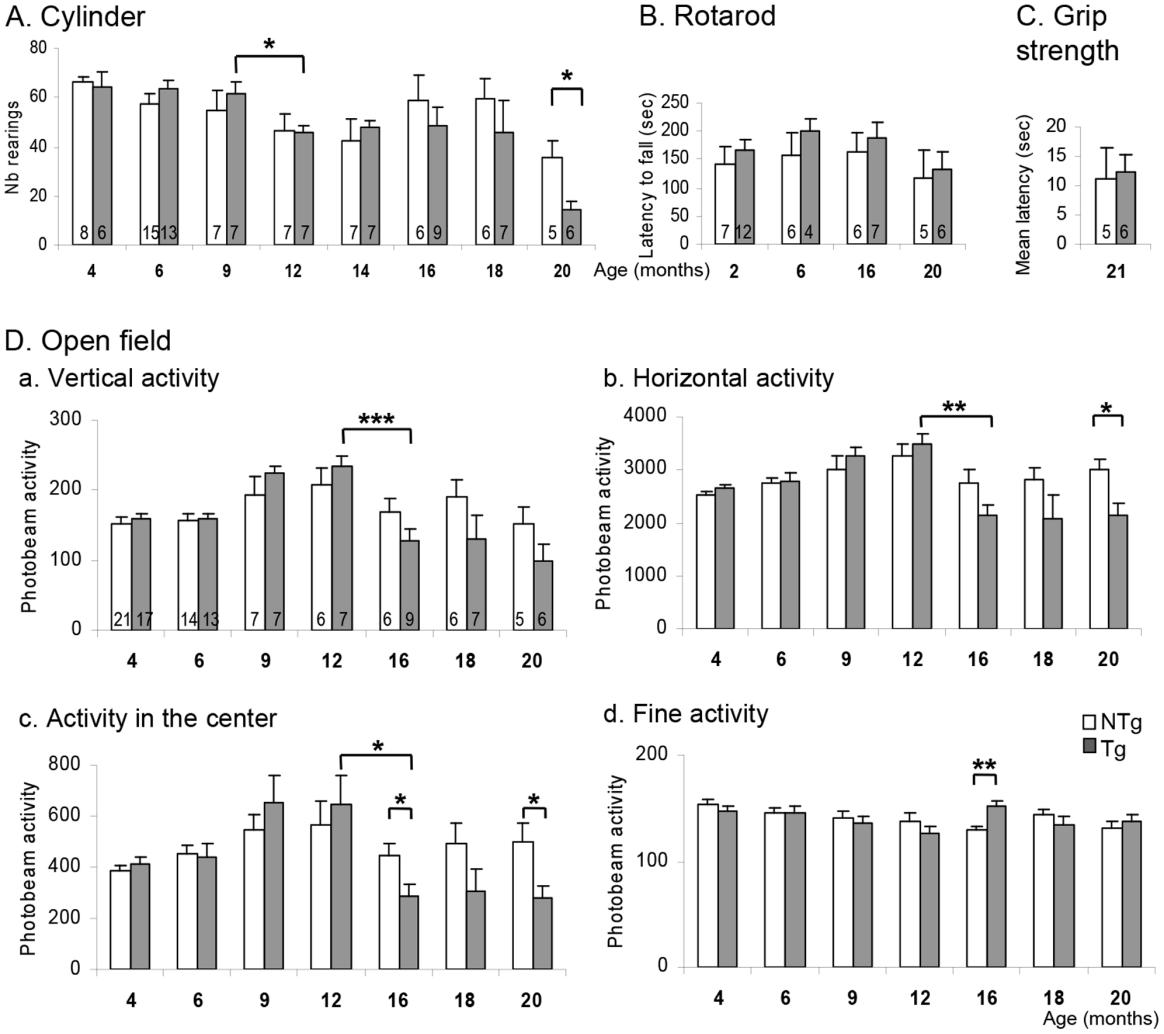
Motor abilities of the BAC *LRRK2^R1441G^* Tg and NTg mice with age. **A.** Number of rearing in the cylinder test. **B.** Latency to fall in the accelerated rotarod paradigm. **C.** Mean latency of staying gripped to the grid of a reverted cage lid. **D.** General activity in the open field test in a 15 min test: rearing (a), horizontal activity (b), activity in the center of the apparatus (c), and total number of fine activities (d). Data are means ± SEM, number of animals are indicated in the corresponding bar of the histogram. *p<0.05, **p<0.01, ***p<0.001 with MANOVA followed by post-hoc comparison (Bonferroni), and Student t test for single comparisons or Welch’s t test when variances were unequal.

As motor symptoms are considered the hallmark of PD in human patients, we first identified the onset of motor disabilities in *LRRK2^R1441G^* mice. Tg and NTg (non-transgenic) littermates coming from several litters were exposed to different tasks measuring locomotion, vertical activity, balance and coordination. Compared to their age- and gender-matched controls, Tg mice displayed subtle motor deficits only after the age of 16 months, though no gross dysfunction could be observed. Indeed, in the standard cylinder test, both groups of mice reared the same number of time within the 5 min session from 4 to 16 months old, but at twenty months old, Tg mice reared much less than their controls NTg (Welch’s t test, t(6.036) = 2.783, p<0.05, **Figure 1A**). Tg mice were still well coordinated in their movements as they performed as good as their controls in the accelerated rotarod (**Figure 1B**). In order to check any decrease of limb muscular tonus that could be responsible for the decreased vertical ability observed in the cylinder test, we measured the ability of the mice to remain clinging to an inverted cage lid (grip strength test, **Figure 1C**). No difference between Tg and NTg could be found concerning the mean latency until they fall from the elevated cage lid, showing that both groups had similar muscular tonus capacity and grip strength at this age.

To measure the spontaneous activity, mice were put in the open field apparatus during 15 min (Photobeam Activity System, PAS-Open Field, San Diego Instrument, USA). Horizontal and vertical activity was automatically recorded as well as the fine activity, which combined actions like grooming, exploration on four limbs and sniffing. We first considered the total amount of activity within the 15 min test (**Figure 1D**). In general, mice increased their activity at 9 months old, but decreased it by half at 16 months old (Age effect with MANOVA with “age” and “genotype” as in-between subject factors, Vertical activity: F(6,131) = 7.066, p<0.001; Horizontal activity: F(6,131) = 6.123, p<0.001). These changes were statistically significant only for Tg animals (Genotype effect with MANOVA with “age” and “genotype” as in-between subject factors, Vertical activity: n.s.; Horizontal activity: F(1,131) = 4.622, p<0.05; followed by post-hoc comparisons, **Figure 1D** **a and 1Db**). From 16 months old, Tg mice were constantly less active than NTg controls, with a significant difference reached at 20 months old (Welch’s t test, t(8.975) = 8.646, p<0.05). A similar profile of activity was observed over time concerning the amount of photobeam activity in the center (MANOVA with “age” and “genotype” as in-between subject factors, Age effect: F(6,131) = 5.239, p<0.001, Genotype effect: n.s., **Figure 1D** **c**), and from 16 months old, the Tg mice spent significantly less time than the controls in the center (Welch’s t test p<0.05 at 16 and 20 months old, with t(11.805) = 5.418 and t(7.550) = 6.143 respectively).

In summary, Tg animals decreased their activity in the open field compared to NTg littermate controls after the age of 16 months old. This was not compensated by an augmentation of fine behaviors. Indeed, although 16 months old Tg mice increased their fine activities compared to NTg (p<0.05, **Figure 1D** **d**), the photobeam activity reflecting the number of fine behaviors were not changed otherwise, suggesting that Tg mice displayed an overall diminution of locomotor activity only.

**Figure 2:**
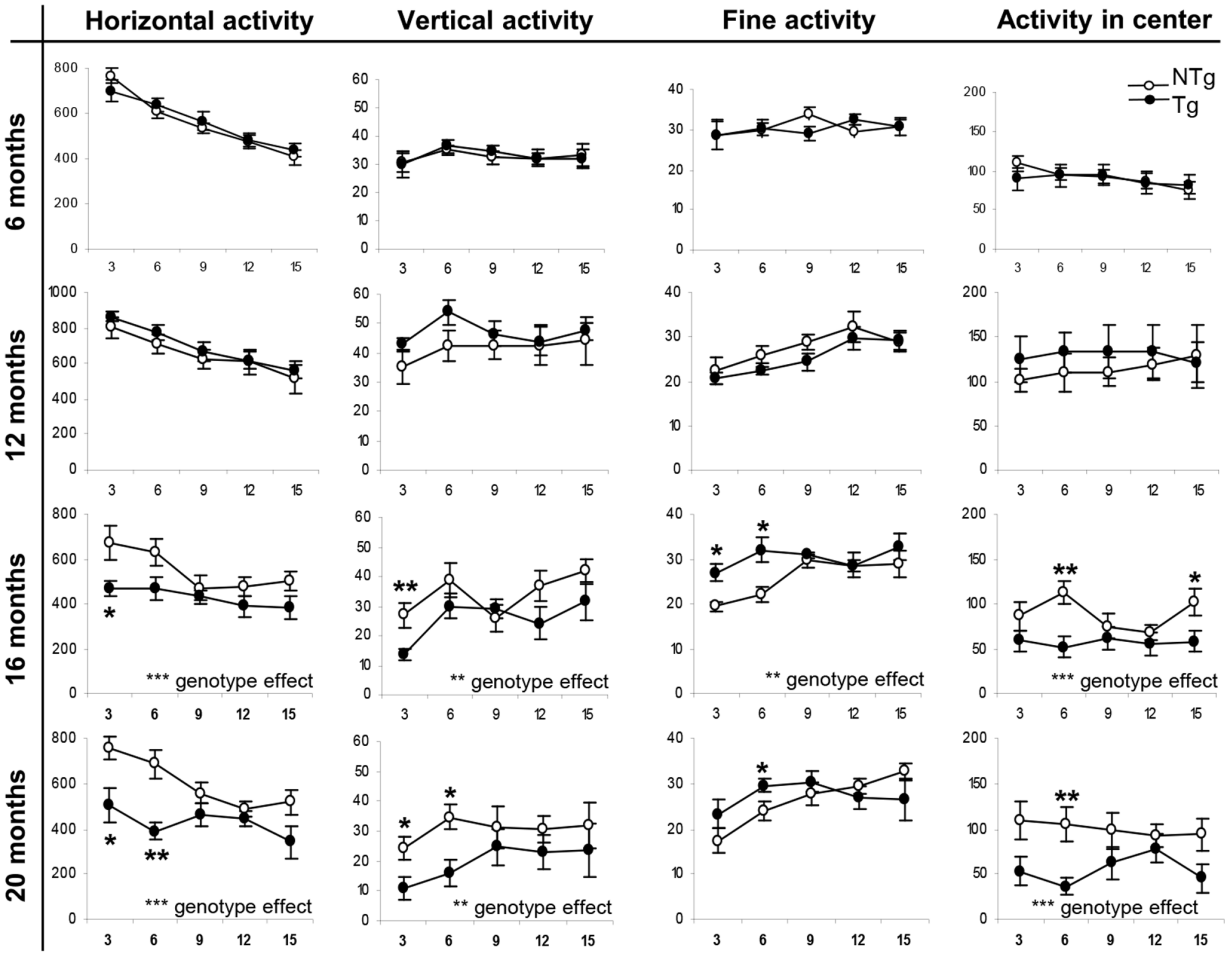
Activity with age in 3 min intervals in the 15 min Open Field test. Horizontal, vertical and fine activity as well as total activity in the center of the arena, of mice at 6, 12, 16 and 20 months old. These results come from the experiments shown in Figure 1 but expressed in 3 min intervals. Data are means ± SEM. *p<0.05, **p<0.01, ***p<0.001, with Welch’s t test within two values of the graph, MANOVA test analysis with “genotype” and “intervals” as factors when considering the genotype effect.

We further compared the activity of both groups according to intervals of 3 min time per 15 min session (**Figure 2**). While the overall activity curves of the mice were similar for both NTg and Tg before one year old, Tg animals showed constantly less horizontal ambulation and rearing behaviors than NTg mice from 16 months old (MANOVA with “genotype” and “3-min-intervals” as in-between subject factors, main effect of the genotype in Horizontal activity at 16 months old: F(1,74) = 14.233, p<0.001, 20 months old F(1,54) = 24.032, p<0.001; and in Vertical activity at 16 months old F(1,74) = 7.811, p<0.01 and 20 months old F(1,54) = 8.565, p<0.01). As previously shown, Tg mice increased their fine movements at 16 months old compared to their NTg littermates (MANOVA with “genotype” and “3-min-intervals” as in-between subject factors, main effect of the genotype: F(1,74) = 10.118, p<0.01). This resting behavior was however not different at older ages, and thus cannot compensate the general decreased activity observed. Also, this analysis confirmed that 16 months old and older Tg mice behaved less in the center of the arena compared to controls (MANOVA with “genotype” and “3-min-intervals” as in-between subject factors, Genotype effect: 16 months old: F(1,74) = 14.617, p<0.001, 20 months old: F(1,54) = 17.874, p<0.001). The results were similar if we considered the percentage of photobeam activity over the total photobeam activity (data not shown, MANOVA with “genotype” and “3-min-intervals” as in-between subject factors, Genotype effect: 16 months old: F(1,74) = 7.678, p<0.01, 20 months old: F(1,54) = 9.375, p<0.001), suggesting that this might not reflect only a reduced locomotor capacity but rather a centrophobic response due to a possible fear of open areas. Since this can be an indicator of anxiety level, we analyzed the mice in other tasks which are sensitive to mood disorders-like behaviors.

### *LRRK2^R1441G^* Mice did not Display Anxiety or Depression-like Behaviors with Age

**Figure 3:**
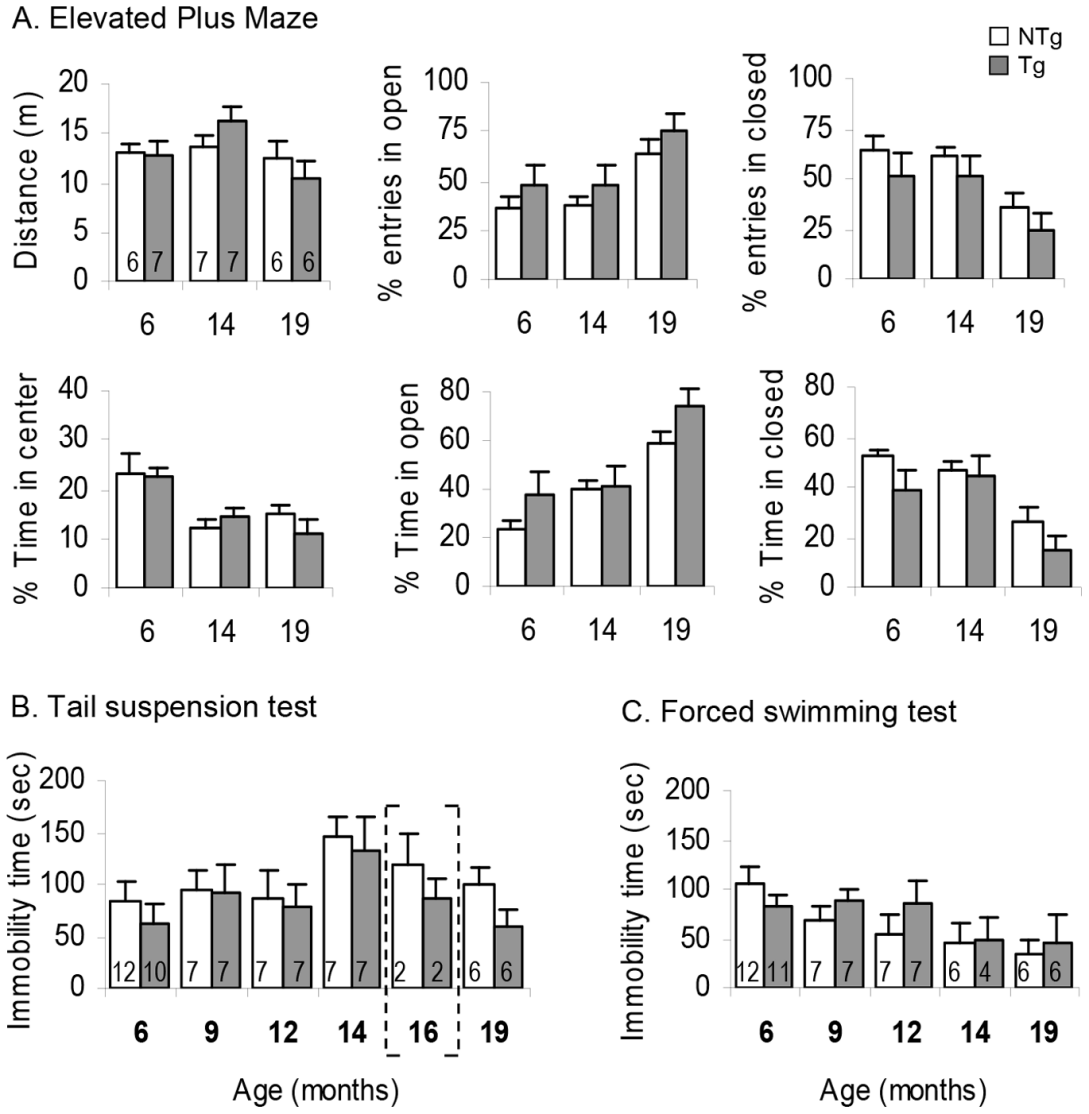
Anxiety and depression-like behaviors in BAC *LRRK2^R1441G^* Tg and NTg mice with age. **A.** **** Distance, percentage of time spent in the opened and closed arms, and percentage of entries done in the opened and closed arms in a 5 min test on the elevated plus maze. **B.** Immobility time recorded in a 6 min tail suspension test. **C.** Immobility time recorded in a 6 min forced swimming test. Data are means ± SEM, number of animals are indicated in the corresponding bar of the histogram. The brackets indicate data given only as indication; these data were not included in the statistics due to the small size of the group considered.

Tg and NTg littermates were tested at 6, 14 and 19 months old in the elevated plus maze. This task measures the willingness of the mice to explore opened and closed areas and informs on the level of anxiety-like behavior of the animals in a new environment. We did not observe any tendency of Tg mice to be more anxious than the NTg (**Figure 3A** **)**. The distance run was similar across ages and genotypes in this 5 min task and the time spent in the opened arms augmented regularly from 6 to 19 months old suggesting an increased exploratory behavior of all mice with age.

Mice were then tested in the tail suspension and forced swimming tests. These tests evaluate the motivation of the animals to escape from a despaired situation, and reflect the depressed state of the animal as compared to the depression-like behavior displayed by humans. Different batches of NTg and Tg mice were tested at different ages from 6 to 19 months old in both tests (**Figure 3B–C** **)**. They all behaved the same, though the Tg mice were slightly more active (i.e. less immobile) than their controls from 16 months old onwards in the tail suspension test (mean immobility time at 16 months old NTg = 118.01±29.99, Tg = 85.91±18.69; at 19 months old NTg = 98.68±16.88, Tg = 60.42±14.35, ANOVA test with “age “ and “ genotype” as in-between factors, Age effect: F(4,76) = 2.539, p<0.05, Genotype effect: F(1,76) = 0.101, n.s.). This was in accordance with the increased exploratory activity found in the elevated plus maze. Similar results were obtained in the forced swimming test with a constant slight decrease of the immobility time with age, but no difference between the two genotypes (ANOVA test with “age” and “genotype” as in-between factors, Age effect: F(4,73) = 1,695, n.s., Genotype effect: F(1,73) = 0.337, n.s.).

### *LRRK2^R1441G^* Mice Displayed Normal Sensory Responses at Late Ages

**Figure 4:**
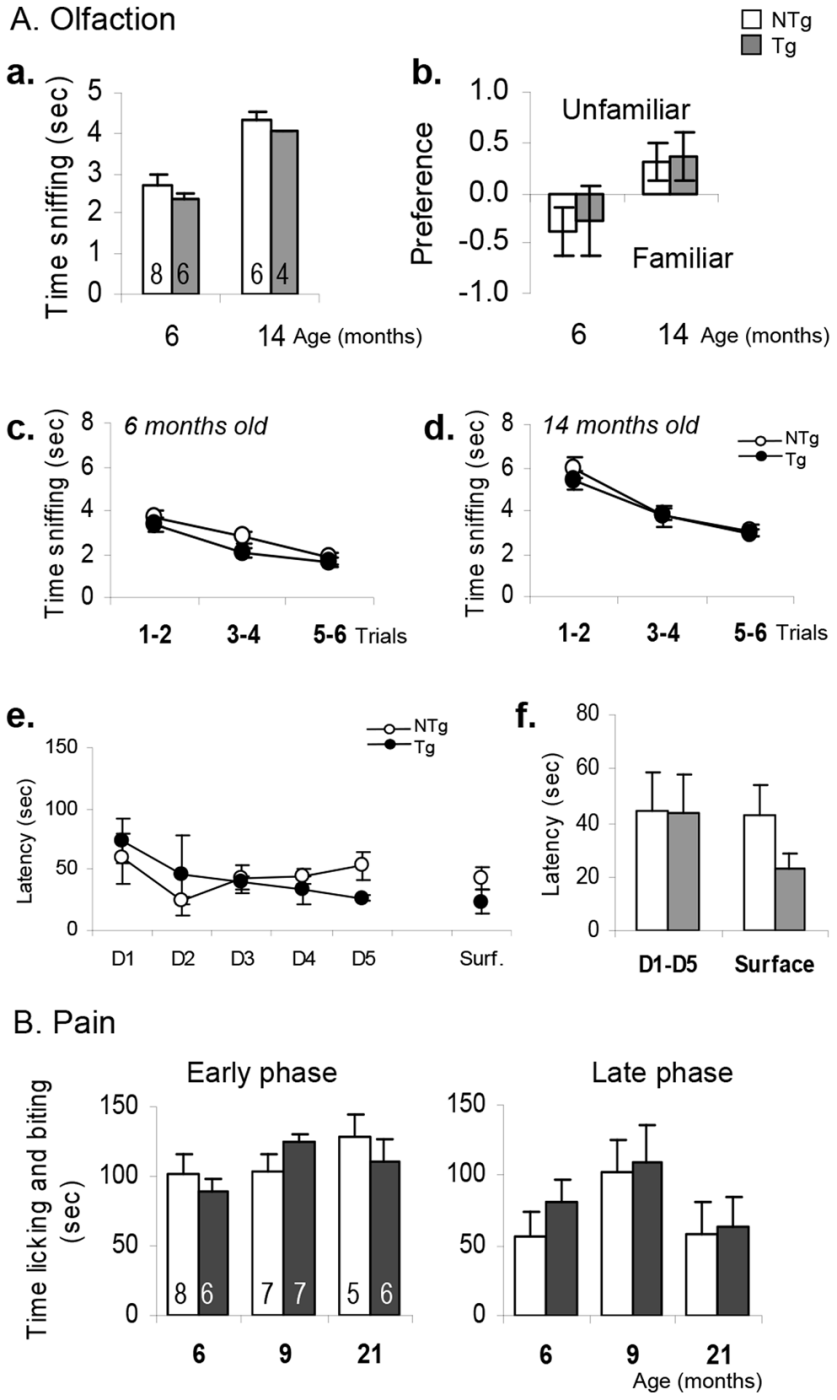
Sensory functions of *LRRK2^R1441G^* Tg and NTg mice with age. A. Olfaction tests at 6 and 14 months old. **a-d.** Wooden block test average time sniffing the familiar block in trial 1 to 6 (a), block preferences in trial 7 (b), and time sniffing the blocks within trials at 6 months (c) and 14 months (d). **e–f.** Latency to find and begin to eat the hidden food within trials in 8 NTg and 6 Tg mice aged 6 months old, and (e) average of latencies to find and eat the hidden food in the same mice (f). “D1” to “D5” represent “Day 1” to “Day 5” and “Surf” or “Surface” means “Surface test” occurring on the 6^th^ day of testing. **B.** Time licking and biting the injured paw in the early and late phases of the 30 min formalin test in mice at 6, 9 and 21 months old. Data are means ± SEM, number of animals are indicated in the corresponding bar of the histogram.

To determine whether the overexpression of the R1441G mutant form of human *LRRK2* gene would affect sensory functions in mice, we checked olfaction abilities with the buried and the block tests, and sensitivity to pain with the formalin test. The two olfaction tests did not reveal any impairment in the ability to smell and recognize either social odors (**Figure 4A** **a-d)** or food odor following deprivation (**Figure 4A** **e-f)**. In the block test, where mice should recognize a familiar odor in scented wooden cubes, Tg and NTg mice tested at 6 and 14 months showed the same pattern independently of their genotype. Interestingly, mice were more interested in sniffing the familiar blocks at 6 months old, whereas 14 months old mice had a preference for the unknown congener’s odors (**Figure 4A** **b)**. No difference between NTg and Tg was detected in the habituation period of the 6 first trials in contact with the blocks scented with the familiar odor of the home cage. In the buried test, 6 months old mice smelled very well the odor of the food following food deprivation, independently of the genotype. Tg mice were not impaired, and were even faster in eating the piece of food in the control trial than the NTg littermates (surface trial, mean latency to find and eat the piece of food NTg = 42.98±9.20; Tg = 23.21±10.00; **Figure 4A** **f)**.

To determine if there was any pain-dependent-sensory dysfunction, we performed the formalin test. Mice were injected with 20 microl of 2.5% formalin in the right hind paw and observed for 30 min. The pain response followed a two-phase curve. The first phase occurred immediately after injection and lasted no more than 2 to 4 min; it is considered as an acute response of local pain. The second phase occurred about 10 to 15 min later corresponding to the response of the central nervous system [34]. Different batches of NTg and Tg mice were tested at 6, 9 and 21 months old. All mice showed similar pain sensitivity without any difference in both phases (**Figure 4B** **)**.

### *LRRK2^R1441G^* Aged Mice had Good Learning Abilities

**Figure 5:**
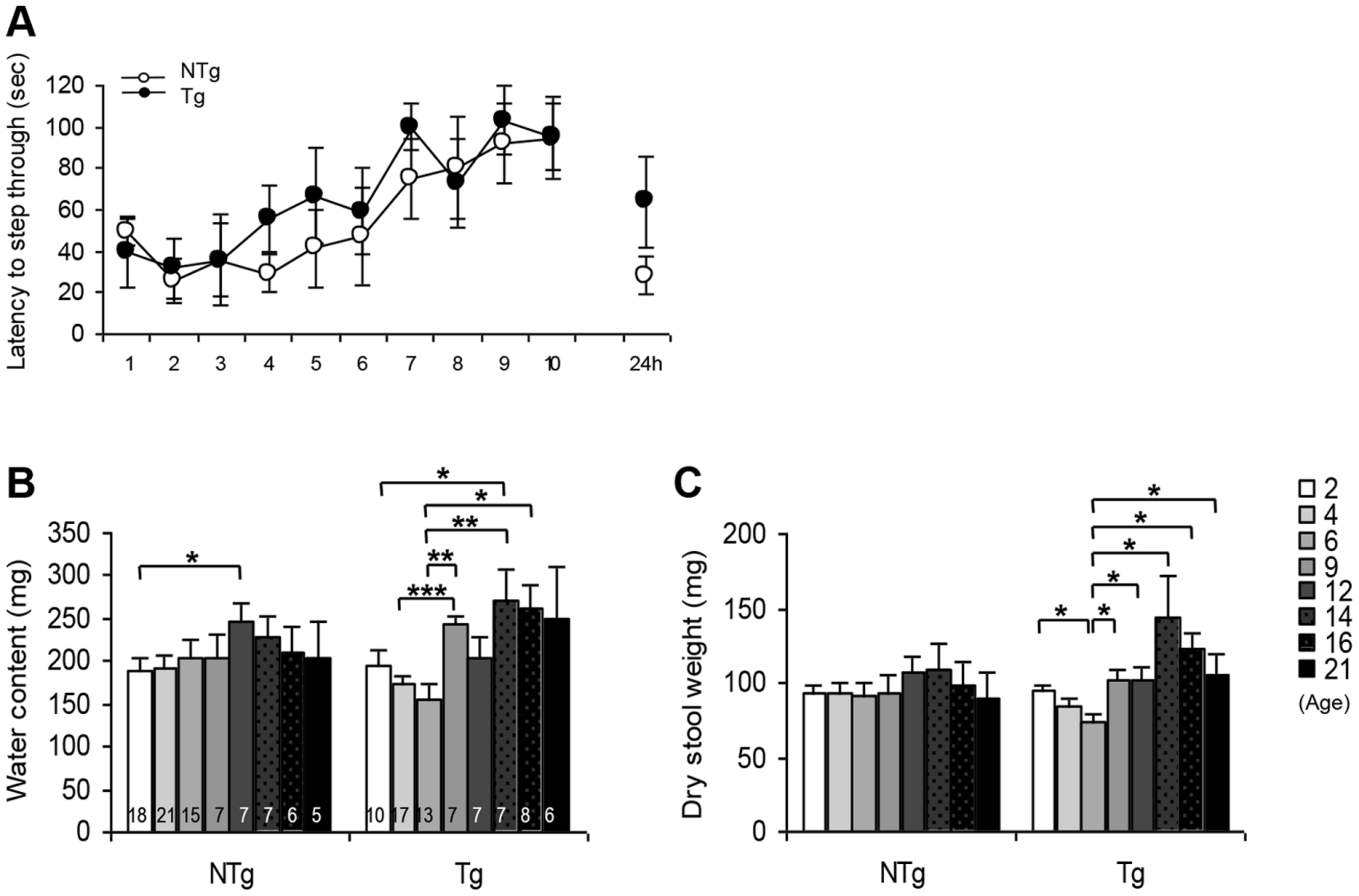
Cognition abilities in the passive avoidance test and gastrointestinal dysfunction in BAC *LRRK2^R1441G^* Tg and NTg mice. **A.** Latency to step through the white compartment with trials in the passive avoidance test. 5 NTg and 6 Tg aged 21 months old were tested up to 10 consecutive times the first day and 24 h after the last trial. **B–C.** Water content in the stool collected in one hour and dry stool weight after evaporation of the water content of Tg and NTg mice aged from 2 to 21 months old. Data are means ± SEM, number of animals are indicated in the corresponding bar of the histogram, *p<0.05, **p<0.01, ***p<0.001 with Welch’s t test for unequal variances.

We then asked whether the *LRRK2^R1441G^* BAC Tg mice displayed learning and memory deficits. Since the sensory function in the paw was not impaired in the formalin test, we tested mice of both genotypes in the passive avoidance task, where the mouse should learn to avoid a mild electric shock given through the grid floor by inhibiting its behavior to enter in the preferred dark compartment. Twenty-one-months old NTg and Tg mice learned similarly well the task as they increased their latency to step through the dark compartment within trials (ANOVA test with “genotype” and “trials” as in-between subjects factors, main effect of trials F(1,110) = 3.929, p<0.001). No difference could be seen between the genotypes (genotype as factor F(1,110) = 1.218, n.s., **Figure 5A**). The mice were retested 24 h after the last trial to check the retention memory. All the mice had a very poor performance in remembering to stay in the light box in this trial. This result might reflect impairment in long term memory abilities linked to ageing, as it was not dependent on the genotype.

### *LRRK2^R1441G^* Mice Displayed Gastrointestinal Dysfunctions from 6 Months Old

Constipation is one of the major problems encountered by PD patients. We thus investigated whether the R1441G mutation in the *LRRK2* gene would have an influence in the stool consistency and compared the production and water content of droppings of NTg and Tg mice at different ages. Although no statistical difference between NTg and Tg animals could be observed at any ages, the overall dropping analysis indicated that Tg mice displayed gastrointestinal problems. While the NTg mice had a constant stool consistency over their life time (ANOVA test comparison with “genotype” and “age” as factors, main Age effect for Water content: F(7,78) = 0.649, n.s.; for Dry Stool Weight: F(7,78) = 0.324, n.s.), Tg animals displayed differences in the water content and weight of dry stool collection from 6 months old, indicating gastrointestinal dysfunctions that oscillate between constipation and diarrhea (ANOVA test comparison with “genotype” and “age” as factors, Age effect for Water content F(7,67) = 2.928, p<0.05; for Dry Stool Weight F(7,67) = 3.776, p<0.01). At 6 months old, Tg mice were clearly constipated. The amount of water in their stool and the total dry stool weight was statistically lower compared to the baseline described at 2 months old. In comparison, the droppings of NTg animals contained a stable amount of water and dry stool from 2 to 9 months old (baseline at 2 months old dry stool weight: NTg = 92.81±5.37, Tg = 93.94±4.48; 6 months old: NTg = 91.18±8.07, Tg = 73.50±6.26, **Figure 5B–C** **).** As Tg mice were getting older, the amount of water in the stool increased, coming back to “normal” (at 9 and 12 months old) then higher compared to the initial baseline, with a maximum at 14 months old (Welch’s t test analysis not detailed here but shown in the graphs with *p<0.05, **p<0.01, ***p<0.001, **Figure 5B–C**).

## Discussion

In a longitudinal study, we have demonstrated that the overexpression of the R1441G mutated form of the human *LRRK2* gene induced mild motor deficits as well as gastrointestinal dysfunctions in mice aged from 4 to 21 months. However, apart from gastrointestinal dysfunctions, it did not trigger major changes in a wide range of non-motor phenotypes, including smell and pain sensitivity, mood disorders-like behaviors, and cognition impairments.

The motor abilities of the *LRRK2^R1441G^* Tg mice were assessed in a battery of tasks measuring horizontal and vertical ability, grip strength, balance and coordination. Compared to age and gender-matched control littermates, the horizontal and vertical activity of Tg mice was decreased from 16 months old onwards in the open field (**Figure 2** **)**, and at 20 months old in the cylinder test (**Figure 1** **).** However, the Tg mice had a good coordination in the rotarod test, and had a grip strength similar to the NTg littermates. The motor impairment was thus restricted to a decrease in horizontal and vertical activity, appearing at an advanced age comparable with the late onset of motor dysfunctions observed in human PD patients.

Though very similar to the knock-out mouse model for the *LRRK2* gene published by Hinkle and coll., [30], these results are not in accordance with those reported by Li and coll. [22]. They showed that the *LRRK2^R1441G^* Tg mice decreased their rearing activity in the cylinder test from the age of 6 months old. At 10 to 12 months old, the Tg mice were heavily impaired and showed a complete akinesia. Although these phenotypes were very convincing (supporting documents with videos in [22]), we were unable to reproduce their results. We believe that animal housing, breeding or other experimental differences are not alone able to explain such discrepancies. We verified that the mice belonged to the same mouse line, as confirmed by the systematic mice genotyping performed by PCR with specific primers amplifying the transgene, and the verification of the transgene expression by Western Blot analysis (**Figure S1).**

According to the literature, other LRRK2 mouse models have also failed to show specific PD motor deficits. Indeed, mouse lines overexpressing the wild type form or the G2019S mutation of the *LRRK2* gene displayed hyperactivity and increased motor function [24], [27], while other lines overexpressing the same G2019S mutation or knock-in for the R1441C mutation did not exhibit particular motor disturbances [24], [25], [31], apart from increased thigmotaxis (preference for the area close to the walls) [23]. Similarly, knock-out mice for the *LRRK2* gene displayed only increased thigmotaxic behavior in the open field and increased abilities in the rotarod [30]. No strong impairment has been reported to date, which is in favor with the idea that *LRRK2* may not be entirely responsible for the motor dysfunction observed in PD patients. Moreover, these lines did not present dopaminergic cell loss, but only impaired dopaminergic neurotransmission, which might explain why the motor functions were only mildly impaired.

Although the R1441G mutation of the *LRRK2* human gene may have little effect on the development of motor features, it might regulate other physiological systems, as shown by the numerous non-motor symptoms associated to PD in humans [36], [37]. Indeed, Tg mice displayed a centrophobic behavior in the open field (or increased thigmotaxis), shown by the decrease in time spent in the center of the open field arena compared to control littermates (**Figure 2** **)**. This could be due to an augmentation of fear or anxiety-like behavior. However, no difference was observed between naive NTg and Tg animals at 6, 14 and 19 months in the elevated plus maze, a test assessing anxiety-like behaviors. At this latter age, Tg mice were even more exploratory than the NTg (% time in open arms at 19 months NTg = 58.41±5.37 and Tg = 73.74±7.53, n.s., **Figure 3A**), though not more active (distance run in 5 min NTg = 12.55±1.53 m, Tg = 10.31±1.77 m). Although the centrophobic response observed in the open field is typically associated to an increase in anxiety-like behavior, it might also be a lack of motivation to explore the new environment, which could be linked to a depressive-like state. However, by testing the animals in despair tests, we couldn’t find any tendency of the Tg mice to adopt the typical depressive-like behavior (to become immobile in the tail suspension or forced swimming test) compared to their controls. In contrast, Tg mice aged 16 months and more were even more active in the tail suspension test compared to NTg littermates (immobility time of Tg was slightly decreased compared to the immobility time of NTg mice, **Figure 3B** **)**.

The motor dysfunctions of the *LRRK2^R1441G^* BAC Tg mice might be attenuated by the high level of activity observed in this mouse line in several tasks. This hyperactivity might be due to the genetic background as FVB mice are known to have a spontaneous high activity compared to other strains [38], [39]. FVB/NJ mice are also homozygous for the retinal degeneration allele *Pde6b^rd1^*, resulting in blindness by wean age (http://jaxmice.jax.org/strain/001800.html). Due to this visual defect, FVB mice might develop stronger senses such as smell or touch, and any sensorial dysfunction similar to PD patients might be easily detectable. In our experiments, Tg and NTg had similar abilities to recognize familiar mouse odors in the block test or find hidden food in the buried test, in accordance with the literature [40]. Six-months old Tg mice were even faster to grab and eat the food than their littermate NTg controls in the probe trial of the buried test (**Figure 4A** **f)**. This could be due to a higher reactivity (faster in performing the test), as there were no evidence that the olfactory function was better. Interestingly, at the same age, all the mice preferred to explore a wooden block scented with familiar home-cage odor rather than a block impregnated with the odors of unknown congeners. The preference was reversed at 14 months old, where mice were more interested in exploring the block scented with different animal beddings, which is the result expected from a normal adult mouse [41]. In summary, the R1441G mutation had no particular effect on the olfactory response of the mice. In fact, this correlates with clinical studies since patients carrying the R1441G mutation do not display main olfactory deficits, in contrast to the majority of PD patients [4], [29], [42], [43].

Similarly, Tg mice did not show any difference in pain sensitivity. The formalin test assesses the way an animal responds to moderate continuous pain generated by injured tissue. It is characterized by two different neurological responses towards the pain stimuli: (i) a first phase, starting immediately after injection of formalin, and responding to the local stimulation of nociceptors, and (ii) a second phase occurring later in time and reflecting the response of the central nervous system towards the stimulus, especially the spinal cord. PD patients display chronic unexplained pain and a decrease in pain tolerance [37], [44]. We therefore expected the Tg mice to show an increase of the pain sensitivity following the injection of the formalin in the paw, and in particular in the second phase, which is considered as a chronic response [34]. However, Tg animals tested at 6, 9 and 21 months of age were not impaired in both phases, suggesting that R1441G mutation do not act on pain dysfunctions. Like many phenotypes, the responses observed in the formalin test are regulated by the genetic background [45], [46], [47]. Since FVB/NJ mice have a greater sensitivity to injuries in general [38], any susceptibility to pain displayed by the Tg mice should thus be seeable. A short study measuring the tactile reaction of the mice when a piece of adhesive tape was put between their ears did not detect any difference between NTg and Tg mice in the latency to remove the tape (data not shown). This corroborated the results observed in the olfaction and pain test and confirmed the absence of any sensory deficits in these mice, even at older ages.

Cognitive impairments in PD range from mild forms of cognitive dysfunction to overt dementia. Although not all patients develop these symptoms, they may arise at late stages of the PD course and are usually associated with more rapid progression of PD disability [37], [48]. From our study, *LRRK2* might not play a role in cognitive dysfunctions, in particular working memory. *LRRK2^R1441G^* mice showed indeed similar learning abilities than the NTg littermates in the passive avoidance test at 21 months old. Both groups of mice have learnt well to avoid the electric chock within 10 trials at an old age, strongly suggesting that these mice will not develop any working memory impairment due to the transgene. Their impairment in visual acuity did not disturb their performance in this test since they could learn well. It has been indeed shown that they have enough visual sensitivity to distinguish a dark area from a bright lighted one [49]. Yet, the good performances of the mice in the passive avoidance test do not allow us to conclude about the possible role of the R1441G mutation on cognitive abilities. Further tests should be conducted to see whether the *LRRK2^R1441G^* mice are impaired in other kind of tasks that are more related to Parkinsonian phenotypes, i.e. procedural- and associative-learning abilities characteristic for striatal impairments. Furthermore, as mentioned before, a large number of tests could not be conducted in these mice due to their genetic background. By breeding the line with another strain to remove the homozygosity of the allele *Pde6b^rd1^*, other tests like the two-object recognition tasks, mazes involving spatial recognition, or the five-choice serial reaction time task for attention deficits could be assessed, for a better cognitive characterization.

One of the clearer phenotypes displayed by the *LRRK2^R1441G^* Tg mice was gastrointestinal dysfunctions. The consistency of the droppings observed in the one-hour stool collection test was deregulated over ages, beginning with a constipation state at 6 months old. This follows the symptoms described in clinical studies, as one of the most common problems observed in PD patients (including patients carrying the R1441G mutation) are gastrointestinal and urinary symptoms [36], [50]. Constipation appears at an early age and other dysfunctions of the digestive system occur frequently at all stages of the disease, such as bowel movements, or even colon or rectal cancers [37].

In conclusion, we have shown that overexpression of the R1441G mutation of the human *LRRK2* gene in mouse did not cause significant behavioral changes (fine behaviors, olfaction, pain sensitivity, mood disorders or cognitive impairments) apart from gastrointestinal dysfunction from 6 months old followed by mild motor deficits at older ages. The observations on olfaction and gastrointestinal function in this model validate findings in human carriers. Compensatory mechanisms modulating the progression of PD at late stages in these animal models should be further evaluated.

## Materials and Methods

### Animals

BAC *LRRK2^R1441G^* mutant Tg and NTg mice were provided by The Jackson Laboratory (#009604, FVB/N-Tg(LRRK2*R1441G)135Cjli/J) and further bred under the same FVB/NJ background. The transgene segregated heterozygously and the genotype of the animals was determined by PCR on tail samples as advised by The Jackson Laboratory. Food and water were given *ad libitum*. Mice were marked randomly at the ears and were tested in all experiments in the same order according to their identification number (number/cage), following standard blocked randomization of testing and data processing [61]. The experiments and analysis were always performed blind of the treatment and genotype.

This study was carried out at the Animal Research Laboratory/Surgical Science and Research Laboratory located in Tan Tock Seng Hospital and co-managed by Tan Tock Seng Hospital and the National Neuroscience Institute, in strict accordance with the recommendations in the Guide for the Care and Use of Laboratory Animals of the National Institutes of Health. The protocol was approved by the Tan Tock Seng Hospital - National Neuroscience Institute (TTSH-NNI) Institutional Animal Care and Use Committee (IACUC) of Singapore (Ref TNI-11/2/003), and all efforts were made to minimize suffering.

### Behavioral Testing

Tg and NTg male mice were tested at 4, 6, 9, 12, 14, 16, 18, 19, 20 and 21 months old. At each age, different tests were done so that mice were naïve as much as possible, except for the open field and the one-hour stool collection tests that were conducted at all ages. Only one test per day was performed, in 3 h time from 9∶00 AM to 3∶00 PM. No more than 5 tests were conducted on the same group of age, from the less to the more stressful one, conducting one test every other day. The formalin test, forced swimming test, the buried test and the passive avoidance test were considered the most stressful tests. Only one of these tests was performed in one mouse at a particular age, and always as the last test of the series, letting 1 to 3 months rest before the next set of experiments. On the testing day, animals were put in the test room at least 20 min before testing in order to acclimatize, and were systematically weighted before the task. The experimenter was always blind of the genotype when performing the tests. The details of the behavioral tests are described below, respecting as possible the order of the assessment schedule.

#### Elevated plus maze

The grey colored high-tech metal alloy maze consisted of a 60 cm elevated plus-shaped apparatus with four arms (35 cm length×5 cm width), two of them being surrounded with walls of 15 cm height (Ugo Basile, Italy). Mice were gently placed on the central platform facing a closed arm and were allowed to freely explore the maze for 5 min. The apparatus was cleaned immediately after each session with cotton pads wetted with 60% ethanol. The test was automatically analyzed with the ANY-maze™ videotracking system (Stoelting, USA).

#### Open field

Mice were allowed to explore freely a cleaned photobeam activity transparent chamber for 15 min (43 x 43 x 43 cm, PAS Open Field, San Diego Instrument, USA). Each mouse was gently placed in the middle of the arena at the start of the test session. Horizontal and vertical activity was recorded automatically and data were analyzed in intervals of 60 to 180 sec over the 15 min session. A central area (X:4, Y:4 in the PAS Open Field system) was designed in order to detect thigmotaxis (preference to move close to the walls) and anxiety-like behavior related to the time spent in the center of the arena. The number of droppings was recorded at the end of the test. Immediately after each session, the apparatus was cleaned with cotton pads wetted with 60% ethanol.

#### Cylinder test

Mice were put into a transparent Plexiglas cylinder (12 cm diameter, 20 cm high) surrounded by two mirrors allowing the observer to see the animal in all directions. The behaviors were video recorded and the number of rearing in the 5 first min was then analyzed manually on the video with a counter. Immediately after each session, the apparatus was cleaned with cotton pads wetted with 60% ethanol.

#### One hour stool collection

Tests were conducted at all ages between 11∶00 AM and 14∶00 PM as described by [65]. Each mouse was placed in a separate clean cage and observed throughout a 60 min collection period. Fecal pellets were collected immediately after expulsion and placed in sealed tubes to avoid evaporation. Tubes were weighted to obtain the wet weight of the stool, then dried overnight at 65°C and weighted again to obtain the dry weight. Stool frequency, dry stool weight and total water in the stool were calculated.

#### Grip strength

Mice were put on a cage lid placed 10 cm below a table covered by towels. The lid was softly shacked to allow the mouse to grip more intensively the mesh, and slowly inverted. The latency to fall was recorded with a stopwatch (Marble, accuracy of 0.01 sec). Mice underwent 4 trials of a maximum of 2 min with 10 to 15 min intertrial. Results were means of all trials.

#### Rotarod

The protocol used was modified from [62]. Mice were put on a rod of an accelerating rotarod for a maximum of 5 min (3 to 30 rpm, 5 min ramp, Ugo Basile, Italy). The latency to fall from the rotating rod was taken on 2 days, with 4 trials per day and an intertrial period of 15–20 min. The 3 last trials of the second day served as a mean value for locomotor abilities. Immediately after each session, the apparatus was cleaned with 60% ethanol.

#### Tail suspension test

Mice were individually suspended by the tail to a horizontal wooden bar 40 cm from the bench top using an adhesive tape placed approximately 1 cm from the tip of the tail. Typically, mice demonstrated several escape-oriented behaviors interspersed with temporally increasing bouts of immobility. The behaviors were videotaped throughout a 6 min test and the immobility time defined as lack of all movements except for whisker movement and respiration was analyzed with a stopwatch.

#### Forced swimming test

Mice were placed individually in a cylinder (height 25.5 cm, diameter 12 cm), containing 14 cm water maintained at 23–25°C. As for the tail suspension test, mice showed typically escape-oriented behaviors by swimming along the cylinder walls, interspersed with immobility, floating in an upright position and making only small movements to keep its head above the water. Behaviors were videotaped and the 6-min test videos were analyzed by a well-trained experimenter with a stopwatch.

#### Block test

Tests were performed as described in [63]. Individually housed animals were exposed to five wooden cubes (2.5 cm^3^) placed inside the cage for one week, during which the bedding was not changed. On the 8^th^ day, the blocks were removed and placed in a plastic bag with the cage and animal number. The first 6 trials consisted of exposing the animal in a clean cage with four of his own blocks, (changing the blocks in each trial, i.e. ABCD, then BCDE, then ABCE, etc). The 7^th^ trial, test trial, consisted of exposing the animal with 3 blocks from his own cage plus one from the cage of another animal. Each exposure lasted 30 sec with 5 min intertrial. The overall experiment was videotaped, and the time spent sniffing the blocks was recorded. Preference in the last trial was expressed with the following formula: ((time sniffing only the block from unknown cage)-(time sniffing only the blocks from his cage))/(total sniffing time).

#### Buried test

Tests were performed as described in [63]. Animals were food restricted up to 90% of their body weight. The tests begun only when mice had reached a stable weight (after 3 to 4 days). The buried test was performed once a day during 5 days. The mouse was put in a new cage where a piece of honey cereal bar (about 250 mg of Oats’n Honey from Nature Valley® Crunchy Granola bar) was hidden at 0.5 cm of the top of a 3 cm high layer of clean bedding. The latency to dig up and begin to eat was taken. Mice were given a maximum of 5 min to find the food. On the 6^th^ day, the piece of food was put on the surface of the clean bedding and the latency to begin to eat was taken.

#### Formalin test

Mice were shortly placed in a restraint cylinder (type 50 ml Falcon tube which extremity was cut) and injected in the right hind paw plantar surface (i.pl.) with 20 microl of 2.5% formalin (in NaCl 0.9%, equiv. 1.84% of formaldehyde, injection done with a 50 microl Hamilton syringe). They were then immediately placed into a glass cylinder (20 cm diameter), surrounded by mirrors to avoid any obstructed view of the animal. The amount of time spent licking and biting the injected paw and leg was considered as indicator of pain and was recorded in 5 min intervals over 30 min. Each animal was tested only once.

#### Passive avoidance test

The test was performed according to [64]. The passive avoidance apparatus (GEMINI Avoidance System, San Diego Instrument, USA) consisted of a test station divided into two compartment enclosures and grid floor (22.8 x 20.3 x 20.3 cm each), one brightly illuminated with a “cue” light and the other dark. In the acquisition session, the mouse was placed in the start compartment and after a 2-sec orientation phase the door was opened allowing the mouse to freely explore all the apparatus. Once the mouse entered the dark compartment (detected by photobeams) the door was closed and the mouse was punished by a mild foot-shock (3 sec, 0.3 mA). At the end of each trial (lasting max 120 sec), the subject returned to the home cage for a 60 sec inter-trial interval. Mice not entering the escape compartment within 120 sec were arbitrarily assigned a latency time of 120 sec. The session terminated either after two consecutive trials without a stepping-through response within 120 sec, or after 10 trials without completion of this passive avoidance acquisition criterion. When the experimental subject reached the criterion before the 10^th^ trial, a 120 sec latency score was assigned to each trial omitted. The Retention session took place 24 h later, and consisted of one trial not punished by foot shock. The Retention trial ended when the animal either gave the step-through response or remained in the start compartment for 120 sec.

### Data Analysis

The behaviors of the mice were analyzed according to the testing possibilities: the activity was automatically recorded either with the photobeam activity system in the open field (PAS-Open field, San Diego Instrument, USA), or displayed automatically on the rotarod apparatus (Ugo Basile, Italy) and passive avoidance Gemini system (San Diego Instrument, USA), or by means of the ANY-maze® videotracking system (Stoelting, USA) in the elevated plus maze apparatus. Data in the cylinder, tail suspension test, forced swimming test, olfactory tests, and pain test were collected manually with a counter or a stopwatch (0.01 sec accuracy). Statistics were done using SPSS Software, v18.0. Two kinds of analysis were usually done: (i) Analysis of variances by ANOVA test for univariate analysis or MANOVA test for multivariate analysis followed by post-hoc comparisons (Bonferroni), in order to compare the scores/variables with multiple factors, i.e. genotype, age, intervals, and/or trials, and (ii) if adequate, a second analysis comparing the scores between genotypes at a specific age or for the same group at different ages with the unpaired Student’s T-Test or Welch’s t test for unequal variance. Equality of variances was assessed by Levene’s Test. Statistical significance was considered only when p<0.05.

### Supplementary Data

#### Protein extraction and western blotting

Brain tissues were washed with PBS and lysed in RIPA buffer containing phosphatase and protease inhibitors. Protein concentration was determined by Bio-Rad protein assay. Equal amounts of protein (20 microg) were separated by electrophoresis on SDS-PAGE gel, and transferred onto nitrocellulose membrane (Millipore). The membranes were blocked with 5% milk in TBST for 1 h at room temperature, followed by probing with specific primary antibodies in 5% milk in TBST for overnight at 4°C (anti-LRRK2, 1∶1000, #3514-1 from Epitomics, and anti-actin, 1∶5000, #SC-69879 from Santa Cruz Biotechnology). Secondary antibody was diluted in 5% milk in TBST, and the incubation was done for 2 h at room temperature. Protein bands were visualized using ECL detection kit (GE Healthcare).

## Supporting Information

Figure S1 **Expression of the LRRK2 protein in different brain areas.** Representative Western Blots of proteins extracted from the brain of Tg and NTg LRRK2*R1441G BAC mice at 12 months old. Actin is represented as loading control.
(TIF)
