# Supplementary figures and images for: Non-Motor and Motor Features in LRRK2 Transgenic Mice
Source: PLoS One. 2013 Jul 30;8(7):e70249. doi: 10.1371/journal.pone.0070249 (PMC3728021; doi:10.1371/journal.pone.0070249)

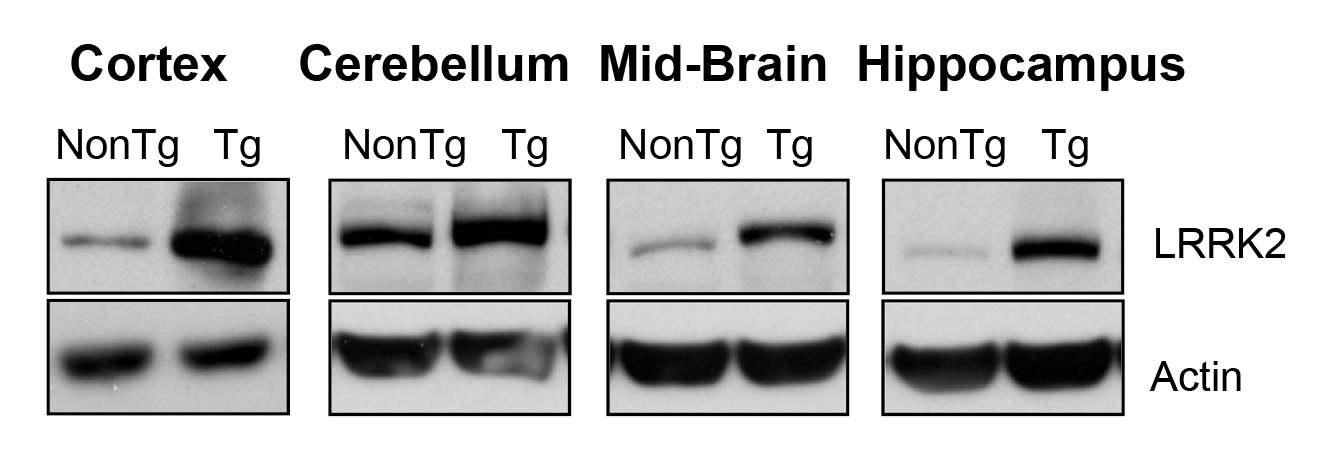

Supplement: Figure S1 — Expression of the LRRK2 protein in different brain areas. Representative Western Blots of proteins extracted from the brain of Tg and NTg LRRK2*R1441G BAC mice at 12 months old. Actin is represented as loading control. (TIF) [file pone.0070249.s001.tif]
